# Seqcrawler: biological data indexing and browsing platform

**DOI:** 10.1186/1471-2105-13-175

**Published:** 2012-07-24

**Authors:** Olivier Sallou, Anthony Bretaudeau, Aurelien Roult

**Affiliations:** 1 Campus de Beaulieu, University of Rennes 1, Rennes, France; 2 INRIA, Campus de Beaulieu, Rennes, France

## Abstract

**Background:**

Seqcrawler takes its roots in software like SRS or Lucegene. It provides an indexing platform to ease the search of data and meta-data in biological banks and it can scale to face the current flow of data. While many biological bank search tools are available on the Internet, mainly provided by large organizations to search their data, there is a lack of free and open source solutions to browse one’s own set of data with a flexible query system and able to scale from a single computer to a cloud system. A personal index platform will help labs and bioinformaticians to search their meta-data but also to build a larger information system with custom subsets of data.

**Results:**

The software is scalable from a single computer to a cloud-based infrastructure. It has been successfully tested in a private cloud with 3 index shards (pieces of index) hosting ~400 millions of sequence information (whole GenBank, UniProt, PDB and others) for a total size of 600 GB in a fault tolerant architecture (high-availability). It has also been successfully integrated with software to add extra meta-data from blast results to enhance users’ result analysis.

**Conclusions:**

Seqcrawler provides a complete open source search and store solution for labs or platforms needing to manage large amount of data/meta-data with a flexible and customizable web interface. All components (search engine, visualization and data storage), though independent, share a common and coherent data system that can be queried with a simple HTTP interface. The solution scales easily and can also provide a high availability infrastructure.

## Background

Labs and bioinformatics platforms have to manage large amounts of data, coming from different sources, in different formats and holding different kinds of meta-data.

**Figure 1:**
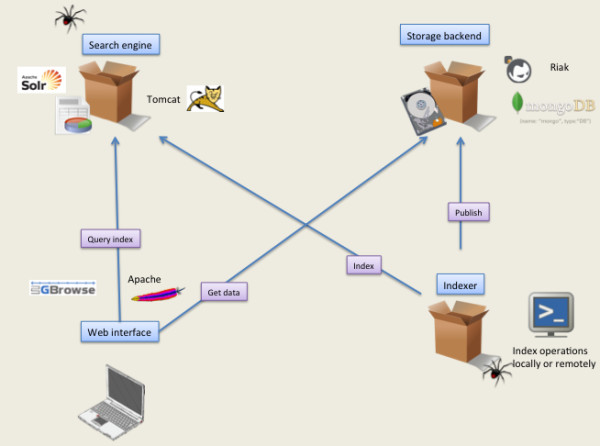
**Components. **Seqcrawler components (indexer, search engine, storage backend, visualization tools) and their interactions.

**Figure 2:**
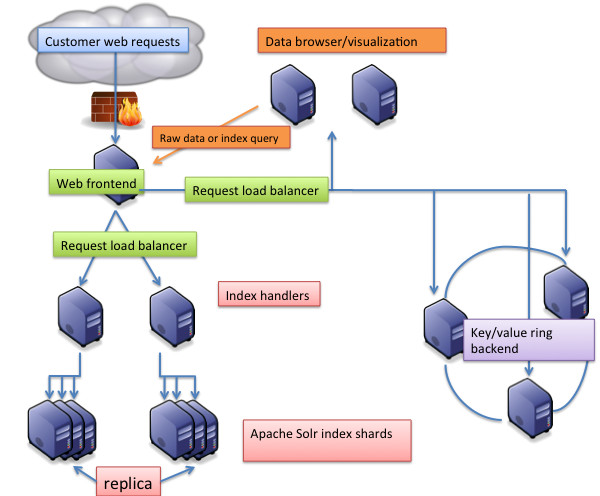
**Architecture and high availability. **The different components can scale independently on one or more servers and can be independently activated or deactivated depending on needed functions. Each component is assigned a different colour. A web front-end can load balance the requests on each component to provide high availability.

While data storage by itself is a new challenge, finding where a specific datum is stored, and what is its meta-data is another one. People need a way to search in their data sets, should it be visually with a graphical interface for analysis, or with an API to integrate it with other applications.

Different tools are available on public bank web sites (EB-eye [1], UCSC [2] or Ensembl genome browsers [3], etc.) to find a specific gene, for example, from its name or ID However those tools are available only for their hosted data; they cannot be mixed up with a specific set of data coming from a lab (except for visualization where a few allow the user to add an extra track from his own dataset, Ensembl for example). Those tools usually limit intensive usage with a risk of being blacklisted. They can prevent automated, frequent requests for large data sets analysis.

The idea was to provide those labs with a tool to access efficiently the data generated in their lab, the same way they would with those online tools, with the additional possibility of mixing them with other data sources. The tool had to be open source, to allow new additional file formats or software extension; flexible, to adapt the web interface to one‘s own need, and scalable to support the load requests and the size of the managed data sets.

Bioinformaticians are the primary target of the project; they need to link their data with the tools they use in their daily tasks. The software gives them access to original document location, the meta-data and some raw data (sequence), all of this in a machine-readable format. A bioinformatician could query the software from a blast result, for example, to extract the features present at alignment positions or he could extract all the information matching a specific criteria. He can also build custom banks from a query matching specific criteria.

The web search interface targets both the bioinformaticians and the scientists for easy access to the information and direct visualization of a set of data (genome browser for example).

The Seqcrawler project started after some testing on Lucegene [4]. Lucegene wrote some base code to link the GMOD GBrowse browser [4] to a search engine (based on Lucene) as well as some translation scripts to ease the indexing of a few biological data formats. However the solution was not scalable to large indexes, and raw data storage was not suitable to hold a very large number of sequences. While SRS [5] offers very interesting features, it is commercial software and does not allow customization or multi-server scalability.

The Seqcrawler goal is to extend those features in an easy to use solution, scalable to fit any need, and implementable in the cloud.

Seqcrawler has been developed to:

1. Enable full text search but also complex queries on meta-data (example: gene position between A and B AND chromosome ID is C).

2. Download original file (record file location and position in file)

3. Provide an integrated, extensible and customisable visualization environment above the indexed data.

4. Provide a search engine and storage back-end scalable on multiple servers

Using a search engine as a central system for the meta-data is interesting for several reasons:

· Search engines provide exact but also approximate text-based search, a useful feature when the user is in front of a search interface.

· Search engines enable storage of information beyond search usage, this means that some data can be stored and accessed even if not used directly for the search.

· Search engines offer scoring and paging results.

· Search engines allow flexible schema, i.e. new fields or document types can be added/indexed with no redefinition.

· GMOD browsers use some drivers to access the data (flat files, SQL…). With an appropriate driver, they can be connected with the search engine.

This work will explain how Seqcrawler can help labs to manage their data and how a biological information system can be built upon it.

## Implementation

### Architecture

The software is made up of several independent components Figure 1.

1. The indexer is a command-line tool to load into the search engine biological data files, from many common known formats (Genbank, GFF3, EMBL, FASTA, PDB, READSEQ [6] supported formats ) and Solr native format.

2. The search engine, Apache Solr [7], manages index queries with a REST- like interface

3. The storage backend, optional, stores sequence data in a key/value based database (MongoDB [8] or Riak [9]). Other backends can be easily added.

4. The web interface triggers the search engine to get results from user query, it manages pagination and provides specific rendering according to original content-type (Gbrowse for GFF/Genbank, …).

The software uploads data (1) and ties the components (2,3,4) to share and extract data. However, each component can be queried or activated independently.

### Components

#### Indexer

The indexer program is a set of Perl/Java command-line tools used to load a document (see supported formats), containing meta-data and/or data (DNA sequences for example), in the search engine and the storage backend. It supports multiple biological formats as input and maps the document to key/value pairs. Each key is defined as a field in the search engine. If indexed, a field can be used as a filter with the search engine. If only stored, it will be included in the answer but will not be part of the filter.

Indexer can index data either offline for large inserts (and better performance), or online on a remote server. The online mechanism can be used for data updates, with no server restart. However, for a very large index, it is advised to update the index in offline mode. The program also supports partial update of the index.

Some formats are managed directly by the software while others are managed with the Readseq software.

The indexer also adds some additional information to keep track of the original file location as well as the location of the data in the file. It also adds a specific content-type to the indexed document, which depends on the original input. This content-type will be used by the web interface to display the data according to its type.

All the search engine fields of the document are indexed by default. This can be customized in the search engine configuration.

While adding a new file format support to the code is quite easy, a plug-in mechanism is provided to dynamically add new ones. To add a new format, the developer writes a new file (myplugin.js), written in JavaScript, with dedicated methods to read the file and transform it in a key/value set. Then, the indexer program can be executed with the new type (−t myplugin) to read the new file format. This plug-in mechanism allows easy integration of new or custom data types.

Another feature is field recoding: a file document field (db_xref of a GenBank file for example) can be recoded (e.g. split, modified) dynamically. To do so, a new Java file can be added and declared in the configuration. This feature helps to customize the parsing of a document.

#### Search engine

The search engine is Apache Solr. It provides a REST-like interface to index a document or query the index. A schema, flexible and customizable, defines how data should be indexed and/or stored for later retrieval. This server supports index shards, i.e. splitting an index into multiple smaller ones while keeping a single point of access to query them. The server manages the querying of all the shards and the merge of the results.

It also supports paging, caching and complex queries on fields with the Lucene [7] syntax. With ranged queries, a field can be queried with upper and lower bounds.

With the REST interface, an application can directly query the system to find an element or a subset of elements (for example, all data with the feature “gene” from chromosome “XXX”).

While providing a filter for each indexed field, the system also supports queries on all the fields with a full text search. Finally, exact but also fuzzy and starts-with searches are allowed.

#### Storage backend

To store large data sets (nucleic sequences for example), in addition to the meta-data, the software uses a key/value based storage backend. Currently designed for MongoDB (default) and Riak, the software can be extended to support other backends.

The reason for such a storage backend is an easy API access and its scalability with a ring architecture. In ring architectures, each node can be queried to get some data, even if the data are not hosted on this node. Data can also be replicated automatically among other nodes to ensure higher availability and performance.

Data are stored as objects with a unique identifier, and can be stored on multiple servers, replicated if needed. This solution provides storage for a very large amount of data elements, with no storage/size limitation.

Datum is split in small chunks in the backend and the application can retrieve it from its ID (chunks will be merged again).

If the original file cannot be accessed by the application, using this storage is a way to keep the data available. It can also be used to store additional data related to the original document (translation field content in a GenBank sheet for example).

The software provides a web interface and a REST- like interface to query the storage backend. It also supports additional start and stop parameters to extract a part of the data (to retrieve a gene from its position in a chromosome for example).

#### Web interface

The web interface is the end-user entry point. This module is not needed for software interaction, as all other components are reachable via a HTTP-based interface. Accessible with any recent browser, the web interface uses Ajax calls to query the index engine and offers export mechanisms to extract all or part of the results. It displays a Google-like result presentation with paging, ordered by score. The search engine calculates the score with internal rules. A few additional meta-data can be displayed depending on the original content type. The display of the results per content-type can be customized (on the server) to add new content-type support or modify the rendering for a specific content-type with the inclusion of a custom JavaScript file.

For each result, additional links are available to:

1. Get the details of a result where all key/value stored elements will be displayed as well as a link to the original document for download.

2. Display the data recorded in the storage backend, depending on content-type.

3. Link to a visualization tool, dependent on content-type.

The content-type (one per supported format, e.g. biosequence/gff for gff based documents), recorded at indexing time by the indexer component, gives some extra information on the original data format. It defines the visualization tool to use (GBrowse for GFF based data, no renderer for UniProt based data, custom ones if any). The software currently embeds 2 visualization tools: GBrowse and ChemDoodle [10].

GBrowse is a well-known sequence browser from GMOD. Linked to the biosequence/gff type (for GFF and GenBank files), it displays the sequence with information extracted from the search engine.

Features information and position are queried via a dedicated DBI interface, and are used by GBrowse to display the information. The DBI interface also queries the storage backend to extract the sequence data to be displayed in the “details” window.

We have developed this specific driver to link the GBrowse software to the Solr engine as an extension of the driver developed by the Lucegene project.

The ChemDoodle renderer is an experimental viewer we have plugged in the system. It displays in the browser a 3D, rotating, image of a protein from a PDB file, in pure JavaScript. This feature needs a recent browser supporting WebGL and is quite CPU consuming.

### Scalability and high availability

The search engine component supports index sharding, i.e. having multiple parts of an index located on different servers but managed as a single, large, index.

The other components are stand-alone components using the search engine to collect the required data.

Each component (1,2,3 and 4, cf. Implementation paragraph) can be scaled and extended on multiple servers to reach expected dimension/load. We could have, for example, 2 search engine shards (2) installed on 2 different servers and a single browser (4) collocated with 1 storage system (3). If the system were to manage additional data after a few months and were in need of additional servers, an additional search engine (2) server could be added and/or an additional storage backend (3) with no breakage on current installation.

The search engine shards can be scaled (and optionally replicated) to add new data on new shards, with no impact on existing shards. One shard (index handler) will query the other shards and merge the results. Each shard can be an index handler.

The data browser /visualization components are independent, as they hold no data/visualization, and can be scaled to face load requests (Figure 2).

The storage backend supports a very large quantity of data on a single server (hundreds of GB), and adding new servers can increase its capacity. The data will automatically be dispatched and replicated, with backend support for node failure and multi-master nodes.

To provide high availability and dispatch the requests to the components, a web load balancer can be set in front of the other servers, balanced per component.

## Results and discussion

The software is successfully installed and used on the GenOuest platform, in a private cloud, hosted on 5 virtual servers (6 to 8 GB RAM, 2 CPU). Several public data banks (genomes from GenBank at NCBI [11], protein data structure from PDB [12], bacteria genomes from NCBI [13], and protein database from Swiss-Prot and UniProt [14]) are indexed for more than 400 millions of records.

The current implementation manages 3 index shards, 1 web dispatcher and 1 server combining storage backend and genome browser.

We have also successfully tested the high availability with duplication of the servers/components and a load balancer.

This high availability test was made with 3 + 3 servers hosting the index shards, 1 + 1 servers hosting the storage backend components with the genome browser software, and one web load balancer. This architecture reduces node failure impact and increases load support.

Documents update, after a remote bank update for example, is automatically managed with the BioMAJ [15] tool, providing a seamless update mechanism.

The software has been integrated with other tools (KoriViewer [16] and other local software) to automate the extraction of additional meta-data from blast results, or to extract the nucleic sequence of a specific id (gene, rnatm, etc.) in the Mobyle portal [17].

Query time can take from 1 to 20 seconds according to the query and the index size. A cache mechanism helps to reduce response times. Each system has to be optimally designed according to the volume of data to manage.

Indexing time also depends on the volume of data. On our platform, using a cluster to parallelize indexing tasks, we index UniProt/Swiss-Prot files in 2 h30.

### Future developments

Expected future developments will add an ontology layer to the data. It is expected to link meta-data and ontologies to be able to extract subsets of data. A query like “get all chromosome identifiers for the species Fish” would match all fish sub-elements in the species ontology. Users of the software will have the possibility of extracting custom banks with a more accurate selection than the current one.

We also expect to add a DAS (Distributed Annotation System) [18] interface to the system to enable DAS compatible tools queries.

## Conclusions

Seqcrawler provides a free and open source solution to meta-data storage and search. While some other solutions on specific web sites will be more accurate, because it is focused on a specific set of data, Seqcrawler offers a flexible solution to locally manage the data, with no restriction on data type and data mixing.

The components of the software are modular, i.e. are component-optional and can be inactivated if not used. Additional components can be introduced to extend the system for other usages; one just needs to query the search engine to extract the meta-data matching one’s criteria.

The system is customizable to fit other requirements, and the plug-in mechanism of the indexer eases the addition of new file formats.

Limitations, from a hardware point of view, are the disk requirements. Index and backend storage require a substantial amount of disk space in addition to the original data. Though, with the sharding support and the ring architecture, data can be split to remove single server disk space restrictions.

The software can scale from single user and small data, up to multi-user and larger data for large organizations. It can also easily be used in a cloud with its packaged installation (Debian/Ubuntu).

## Availability and requirements

The Seqcrawler software is freely available for download with installation instructions at http://seqcrawler.sourceforge.net

· **Project name**: Seqcrawler

· **Project home page**: seqcrawler.sourceforge.net

**· Operating system(s)**: Platform independent

· **Programming language**: Java/JavaScript

· **Other requirements**: Java 1.6 or higher, Tomcat 5.0 or higher

**· License**: CeCILL

· **Any restrictions to use by non-academics**: none

**· Demo**: http://seqcrawler.genouest.org

While the software depends on multiple components, not always easy to install, a deb package is available for Debian/Ubuntu distributions. In case of missing dependency, dependencies are available on Debian mirrors. The package is installed with a sample bank for immediate testing.

Complete installation is detailed on the project web site in the Installation section.

## Abbreviations

Ajax: Asynchronous JavaScript and XML. Techniques used in web programming for asynchronous requests and rendering; API: Application Programming Interface (http://en.wikipedia.org/wiki/Application_programming_interface); DAS: Distributed Annotation System (http://www.biodas.org/documents/spec-1.53.html); DBI: DataBase Interface, i.e. driver to database; GMOD: Generic Model Organism Database project; REST: Representational state transfer (http://en.wikipedia.org/wiki/REST). All requests are available with HTTP GET requests and URL parameters; WebGL: Web based Graphics Library, JavaScript library to use the computer display card's Graphics Processing Unit (GPU).

## Competing interests

The authors declare that they have no competing interests.

## Authors’ contributions

OS developed the software and packaged it. AR installed the infrastructure and made all the system installation on the GenOuest platform. AB helped on the design of the web interface. All authors read and approved the final manuscript.
